# Renal transplantation in older adults: retrospective cohort study to examine the impact of the new 2019 kidney offering scheme on older adult transplant recipients

**DOI:** 10.1308/rcsann.2024.0062

**Published:** 2024-10-08

**Authors:** O Edginton, M George, C Bandara, M Johnston, A Rao, M Howse, D Ridgway, P Goldsmith

**Affiliations:** ^1^ University of Liverpool, UK; ^2^ Liverpool University Hospitals NHS Foundation Trust, UK

**Keywords:** Kidney transplantation, Older age, Elderly, Frailty

## Abstract

**Introduction:**

In 2019, a new kidney offering scheme was launched in the United Kingdom, aiming to better match estimated patient survival and graft life expectancy. The scheme's impact on older patients undergoing kidney transplantation (KT) is unknown. This study aims to compare the outcomes of older adult KT recipients before and after introduction of the 2019 scheme.

**Methods:**

A retrospective observational cohort study of older adults who underwent KT was undertaken. Group 1 were transplanted between 1 September 2017 and 31 August 2019 (2006 allocation scheme) and group 2 between 1 September 2019 and 31 August 2021 (2019 offering scheme). An older adult was any person ≥60 years old at the time of KT. Univariable binary logistic regression analysis was performed to determine odds ratios (OR) and 95% confidence intervals (CI).

**Results:**

There were 107 older adult deceased donor KT recipients, 62 from group 1 and 45 from group 2. Median age at transplantation was 68 (interquartile range [IQR] 62–71) and 67 (IQR 64–73) years, respectively. Univariable analysis showed that re-intervention (OR 6.486, 95% CI 1.306–32.216, *p* = 0.022) and critical care admission (OR 5.619, 95% CI 1.448–21.812, *p* = 0.013) were significantly more likely in group 2. Group 2 recipients were significantly more likely to have a level 4 human leucocyte antigen (HLA) mismatch (OR 4.667, 95% CI 1.640–13.275, *p* = 0.004) and to have undergone previous KT (OR 4.691, 95% CI 1.385–15.893, *p* = 0.013).

**Conclusions:**

The introduction of the 2019 offering scheme was associated with re-intervention and critical care admission for older KT recipients. We also observed less-favourable HLA matches but more KT in difficult-to-match groups.

## Introduction

Kidney transplantation (KT) is the best form of kidney replacement therapy (KRT), exhibiting significantly improved mortality and morbidity compared with dialysis.^1^ In the United Kingdom (UK), the prevalence of patients on KRT is increasing, with 36.8% of affected patients aged ≥65 years.^2,3^ Rates of KT are also increasing, with the United States seeing numbers in people aged >65 years triple between 1998 and 2016.^4^

KT recipients experience improved survival compared with waiting list patients.^5–8^ Uncertainty remains as to whether a survival benefit persists in older adults,^7,9^ and there are concerns that significant surgical intervention could negatively impact morbidity. The complex interplay between donor characteristics, recipient comorbidities and frailty means careful selection for KT is required.^10,11^ Current preoperative assessment focuses on cardiovascular fitness for anaesthesia rather than taking a more holistic approach encompassing frailty assessment.^12^

In September 2019, a new kidney offering scheme was launched in the UK. The previous scheme, from 2006, saw patients from ethnic minority backgrounds and those highly sensitised to human leucocyte antigens (HLA) face disproportionately long wait times.^13^ The 2019 offering scheme classifies recipients into two tiers. Tier A prioritises individuals who are difficult to match immunologically and who have experienced long wait times.^14^ The remaining patients fall into Tier B, which uses calculations to match donors to recipients based on their risk (Supplemental Figure 1, available online). The new scheme prioritises matching of estimated patient survival and graft life expectancy, with more points awarded to donors and recipients who are well risk-matched.^13,14^ Increased emphasis on risk-matching means less-favourable kidneys, termed ‘expanded criteria donor’ (ECD) kidneys, will likely be offered to older transplant recipients.^15^

This study aimed to compare the outcomes of adults aged ≥60 years undergoing KT under the 2006 allocation scheme and the 2019 offering scheme, hypothesising that the new scheme may adversely impact perioperative complications, morbidity and mortality.

## Methods

This study followed the strengthening the reporting of observational studies in epidemiology (STROBE) statement.^16^

### Study population

An observational retrospective cohort study of older adult KT recipients was undertaken at a centre in the northwest of England that performs 100–120 KT per annum.

Older adult recipients, defined as individuals aged ≥60 years at the time of KT, were identified. KRT patients are often comorbid and frail, meaning that chronological age does not accurately reflect physiological age.^4,11^ This study utilised an age threshold of ≥60 years, as applied in other studies, to capture those at risk of geriatric syndromes, given the risk of multimorbidity and frailty within the study population.^17,18^ At our institution, this age threshold is already used for induction immunosuppression; patients ≥60 years old receive an attenuated dose of alemtuzumab because of a local review that identified higher rates of post-transplant Cytomegalovirus (CMV) viraemia in this group.

Eligible patients were assigned to two groups: group 1, KT between 1 September 2017 and 31 August 2019 under the ‘2006 allocation scheme’; and group 2, KT between 1 September 2019 and 31 August 2021 under the ‘2019 offering scheme’. Live donor KT recipients were excluded.

All KT recipients underwent routine cardiorespiratory investigation including electrocardiogram and echocardiogram. When indicated, older adult recipients underwent cardiac myocardial perfusion scan or cardiac stress echocardiogram.

### Outcome measures and definitions

Postoperative outcomes including length of stay (LOS), delayed graft function (DGF) and unplanned critical care admission were collected. DGF was defined as the need for dialysis in the first 7 days post-transplantation. Early graft loss was defined as graft failure in the 30 days after KT.

Follow-up outcomes evaluated within the first year included need for re-intervention, acute rejection, graft biopsy, unplanned readmission (routine readmissions for ureteric stent or peritoneal dialysis catheters were excluded), CMV viraemia and a diagnosis of malignancy. Re-intervention was defined as a return to theatre or interventional radiology procedure. Patients were followed-up to one year post KT. Outcomes were collected from patient electronic clinical records.

### Variables

Patients were identified from local transplant databases. Patient demographics including age at KT, gender, ethnicity, smoking status, primary renal disease, body mass index, comorbidities, modality of KRT before KT, previous KT and dialysis vintage were determined. Comorbidities were analysed using the Charlson Comorbidity Index (CCI) (Supplemental Table 1, available online).^19^ Transplant characteristics including donor type, donor age, ECD transplantation, cold ischaemia time (CIT), calculated reaction frequency (cRF) and donor–recipient CMV mismatch were determined from the local transplant database and medical records. An ECD transplantation was defined as a donor >60 years old or a donor >50 years old with two of: history of hypertension, death by intracranial haemorrhage or creatinine >133µmol/L.^15^ HLA-A, HLA-B and HLA-DR mismatches were recorded and categorised according to HLA mismatch levels (Supplemental Table 2, available online).

### Statistical analysis

Summary statistics utilised frequency and proportion for categorical variables. Discrete variables were analysed using a chi-squared or Fisher's exact test. Continuous variables were expressed using a median with interquartile range (IQR) and were analysed using a Mann–Whitney *U* test. None of the data were normally distributed.

A univariable logistic regression model was used to determine variables that were associated with the 2019 offering scheme. In the model, binary code was used: 1 = 2019 offering scheme and 0 = 2006 allocation scheme. Univariable logistic regression models were run for each of the following explanatory variables of known clinical importance and reported as odds ratios (OR) and 95% confidence intervals (95% CI).^20^ The explanatory variables used included previous KT, Caucasian ethnicity, dialysis vintage, donor age, level 4 HLA mismatch, LOS, CIT, induction immunosuppression, critical care admission, re-intervention, DGF, graft biopsy, acute rejection, CMV viraemia, readmission, and patient and graft survival at one year.

Kaplan–Meier graphs with survival tables and a log-rank test were performed to compare patient and graft survival at one year.

All *p*-values were considered significant at <0.05. Statistical analysis was performed using IBM SPSS Version 26 for Windows.^21^

### Ethics approval

Local trust approval was granted. Patient information was pseudonymised and stored in a password-protected Microsoft Excel database.

## Results

**Figure 1:**
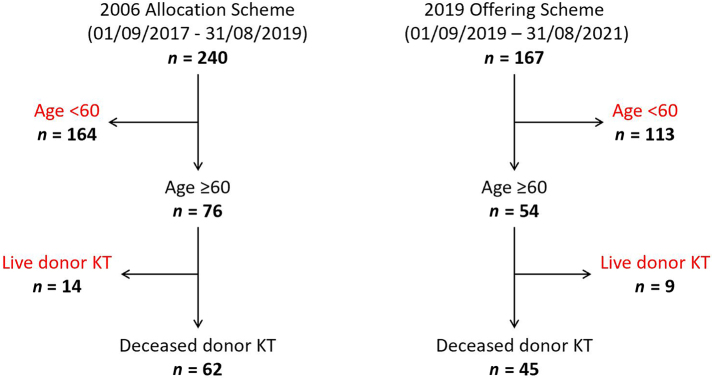
Patient flowchart

A total of 407 patients underwent KT during the study period (Figure 1): 2006 allocation scheme *n* = 240; 2019 offering scheme *n* = 167. Despite the COVID-19 pandemic affecting group 2, the proportion of eligible patients aged ≥60 years old was similar between the 2006 allocation scheme (31.7%) and the 2019 offering scheme (32.3%). Live donor KTs were excluded, leaving 62 patients transplanted during the 2006 allocation scheme and 45 in the 2019 offering scheme.

### Recipient and transplant characteristics

Patient characteristics are summarised in Table 1. Median age at KT was 68 years (IQR 62–71) in the 2006 allocation scheme and 67 years (IQR 64–73) in the 2019 offering scheme (*p* = 0.420). In the 2006 allocation scheme, all but one patient was Caucasian (98.4%). The 2019 offering scheme led to more transplantations in Black, Asian and Minority Ethnic (BAME) groups (11.1%): three patients (6.7%) were South Asian, one patient (2.2%) was Chinese and one patient (2.2%) was African. The median dialysis vintage was 22.5 months (IQR 3.5–39.0) during the 2006 allocation scheme and 18 months (IQR 0–49.5) under the 2019 offering scheme (*p* = 0.858). In the 2019 offering scheme more recipients had undergone a previous KT (6.5% vs 24.4%, *p* = 0.011).

**Table 1:** Patient characteristics

	2006 allocation scheme, *n* = 62	2019 offering scheme, *n* = 45	*p*-value
Median age at KT, years [IQR]	68 [62–71]	67 [64–73]	0.420^a^
Gender			
Male	43 (69.4)	30 (66.7)	0.768^b^
Female	19 (30.6)	15 (33.3)	
Ethnicity			
Caucasian	61 (98.4)	40 (88.9)	0.080^c^
BAME groups*	1 (1.6)	5 (11.1)	
Smoking status			
Non-smoker	31 (50.0)	21 (46.7)	0.062^c^
Ex-smoker	20 (32.2)	18 (40.0)	
Current smoker	4 (6.5)	6 (13.3)	
Not recorded	7 (11.3)	0 (0)	
Modality of KRT			
Pre-emptive	13 (21.0)	15 (33.3)	0.305^b^
Haemodialysis	37 (59.7)	21 (46.7)	
Peritoneal dialysis	12 (19.3)	9 (20.0)	
Median dialysis vintage, months [IQR]	22.5 [3.5–39.0]	18 [0–49.5]	0.858^a^
cRF			
0%	52 (83.9)	33 (73.4)	0.233^c^
1–84%	7 (11.3)	6 (13.3)	
≥85%	3 (4.8)	6 (13.3)	
Previous KT	4 (6.5)	11 (24.4)	**0.011** ^c^
Median Charlson Comorbidity Index [IQR]	5 [5–6]	5 [4–6]	0.124^a^
Body mass index			
18–25	21 (33.9)	21 (46.7)	0.282^b^
25–30	29 (46.8)	14 (31.1)	
>30	12 (19.3)	9 (20)	
Not recorded	0 (0)	1 (2.2)	
Cause of end-stage renal failure			
Diabetes mellitus	10 (16.1)	4 (8.9)	0.903^c^
Polycystic kidney disease	9 (14.5)	5 (11.1)	
IgA nephropathy, FSGS and vasculitidies	14 (22.6)	10 (22.2)	
Drug induced	3 (4.8)	5 (11.1)	
Glomerulonephritis	9 (14.5)	7 (15.6)	
Hypertension	6 (9.7)	6 (13.3)	
Obstructive uropathy	3 (4.8)	1 (2.2)	
Infections	1 (1.6)	1 (2.2)	
Unknown	6 (9.7)	4 (8.9)	
Other	1 (1.6)	2 (4.4)	

BAME = Black, Asian and Minority Ethnic; cRF = calculated reaction frequency; FSGS = focal segmental glomerulosclerosis; IgA = immunoglobulin A; IQR = interquartile range; KRT = kidney replacement therapy; KT = kidney transplantation

Values in parentheses are percentages unless otherwise stated. Statistically significant *p*-values are shown in bold

^a^Mann–Whitney *U* test

^b^Chi-squared test

^c^Fisher’s exact test

*BAME groups include: 2006 allocation scheme – South Asian *n* = 1 (1.6), 2019 offering scheme – South Asian *n* = 3 (6.7), Chinese *n* = 1 (2.2), African *n* = 1 (2.2).

Transplant characteristics are displayed in Table 2. Median donor age increased from 56.5 years (IQR 48–67.25) in the 2006 allocation scheme to 62 years (IQR 50.5–70) in the 2019 offering scheme, as did the rates of ECD transplantation (48.4% vs 57.8%, *p* = 0.337) but neither was statistically significant. Alemtuzumab induction immunosuppression was used less frequently in the 2019 offering scheme (53.2% vs 28.9%, *p* = 0.017). Median CIT increased from 818min [664–1,080.25] in the 2006 allocation scheme to 974min [754.5–1,176.5] in the 2019 offering scheme (*p* = 0.033).

**Table 2:** Transplant characteristics

	2006 allocation scheme, *n* = 62	2019 offering scheme, *n* = 45	*p*-value
Median donor age, years [IQR]	56.5 [48–67.25]	62 [50.5–70]	0.112^a^
Donor type			
DBD	35 (56.5)	27 (60.0)	0.714^b^
DCD	27 (43.5)	18 (40.0)	
Dual transplantation	5 (8.1)	1 (2.2)	0.397^c^
ECD transplantation	30 (48.4)	26 (57.8)	0.337^b^
Median cold ischaemia time, minutes [IQR]	818 [664–1,080.25]	974 [754.5–1,176.5]	**0.033** ^a^
CMV mismatch	16 (25.8)	6 (13.3)	0.148^b^
Induction immunosuppression agent			
Basiliximab	29 (46.8)	32 (71.1)	**0.017** ^b^
Alemtuzumab	33 (53.2)	13 (28.9)	
Eligible for and received alemtuzumab*	31 (81.6)	13 (44.8)	**0.002** ^b^
HLA mismatch level			
Level 1	5 (8.1)	0 (0)	0.072^c^
Level 2	9 (14.5)	7 (15.6)	1.000^b^
Level 3	42 (67.7)	23 (51.1)	0.109^b^
Level 4	6 (9.7)	15 (33.3)	**0.003** ^b^

CMV = Cytomegalovirus; DBD = donation after brain death; DCD = donation after circulatory death; ECD = expanded criteria donor; HLA = human leucocyte antigens; IQR = interquartile range

Values in parentheses are percentages unless otherwise stated. Statistically significant *p*-values are shown in bold

^a^Mann–Whitney *U* test

^b^Chi-squared test

^c^Fisher’s exact test

*Percentages do not reflect column totals. Occasionally, basiliximab was given instead of alemtuzumab owing to donor or recipient factors off usual clinical protocol. This was much more frequent in the immediate recovery from the COVID pandemic when alemtuzumab was not used for some eligible recipients.

HLA mismatch total was calculated by adding HLA-A, HLA-B and HLA-DR to give a score ranging from zero to six as a quantitative descriptor of HLA mismatch (Supplemental Figure 2, available online). The HLA mismatch levels for both cohorts are displayed in Table 2. The least favourable HLA mismatch, termed a level 4 HLA mismatch (1DR and 2B or 2DR), was significantly greater in the 2019 offering scheme (9.7% vs 33.3%, *p* = 0.003).

### Postoperative and one-year outcomes

Patient outcomes are displayed in Table 3. More patients in the 2019 offering scheme required critical care admission postoperatively (3.2% vs 22.2%, *p* = 0.014). Indications included requirement for optimisation of physiological instability (2006 allocation scheme *n* = 2, 2019 offering scheme *n* = 4), and surgical complexity or transplant-related complication (2006 allocation scheme *n* = 1, 2019 offering scheme *n* = 6).

**Table 3:** Patient outcomes

	2006 allocation scheme, *n* = 62	2019 offering scheme, *n* = 45	*p*-value
Median LOS [IQR]	9 [7–12]	10 [7–13]	0.515^a^
Critical care admission	3 (3.2)	10 (22.2)	**0.014** ^b^
Re-intervention	2 (3.2)	8 (17.8)	**0.016** ^b^
Graft biopsy	14 (22.6)	14 (31.1)	0.376^c^
DGF	14 (22.6)	13 (28.9)	0.504^c^
Acute rejection	6 (9.7)	9 (20.0)	0.162^c^
CMV viraemia	6 (9.7)	16 (35.6)	**0.001** ^c^
Fall	5 (8.1)	5 (11.1)	0.739^b^
Malignancy	2 (3.2)	2 (4.4)	1.000^b^
Readmission	27 (43.5)	27 (60.0)	0.118^c^

CMV = Cytomegalovirus; DGF = delayed graft function; IQR = interquartile range

Values in parentheses are percentages unless otherwise stated. Statistically significant *p*-values are shown in bold

^a^Mann–Whitney *U* test

^b^Fisher’s exact test

^c^Chi-squared test.

Significantly more KT recipients required re-intervention in the 2019 offering scheme (3.2% vs 17.8%, *p* = 0.016). The majority (80.0%) underwent re-intervention for indications directly related to KT. Two patients in the 2019 offering scheme group required surgical intervention for indirect complications of KT and its recovery including a complication relating to a fall and one due to surgical complexity.

More patients had biopsy-confirmed acute rejection in the 2019 offering scheme group (9.7% vs 20.0%, *p* = 0.129) (Table 4). Rates of CMV viraemia were significantly greater in the 2019 offering scheme (9.7% vs 35.6%, *p* = 0.001). CMV viraemia was significantly more likely in those patients who had acute rejection in the 2019 offering scheme group (16.7% vs 77.8%, *p* = 0.041). Furthermore, the majority of patients experiencing acute rejection during the 2019 offering scheme received basiliximab (88.9%) induction immunosuppression.

**Table 4:** Comparison of episodes of acute rejection between the 2006 allocation scheme and 2019 offering scheme

	Acute rejection 2006 allocation scheme, *n* = 6	Acute rejection 2019 offering scheme, *n* = 9	*p*-value
Induction immunosuppression			
Basiliximab	2 (33.3)	8 (88.9)	0.089
Alemtuzumab	4 (67.7)	1 (11.1)	
Eligible for alemtuzumab^a^	4 (67.7)	6 (66.7)	1.000
Highly sensitised (cRF >85%)	0 (0)	2 (22.2)	0.486
CMV viraemia	1 (16.7)	7 (77.8)	**0.041**
CMV mismatch	1 (16.7)	0 (0)	0.400
HLA mismatch level			
Level 4	0 (0)	2 (22.2)	0.486
Level 3	3 (50.0)	5 (55.6)	1.000
Level 2	2 (33.3)	2 (22.2)	1.000
Level 1	1 (16.7)	0 (0)	0.400

CMV = Cytomegalovirus; cRF = calculated reaction frequency; HLA = human leucocyte antigens

Values in parentheses are percentages unless otherwise stated. Statistically significant *p*-values are shown in bold. Statistical analysis using Fisher’s exact test

^a^Occasionally, basiliximab was given instead of alemtuzumab because of donor or recipient factors off usual clinical protocol. This was much more frequent in the immediate recovery from the COVID pandemic when alemtuzumab was not used for some eligible recipients.

The readmission rates were 43.5% for the 2006 allocation scheme and 60% for the 2019 offering scheme (*p* = 0.118). A notable number of falls were recorded in the year after KT, although rates were similar between the groups (8.1% vs 11.1%, *p* = 0.739).

### Patient and graft survival

**Figure 2:**
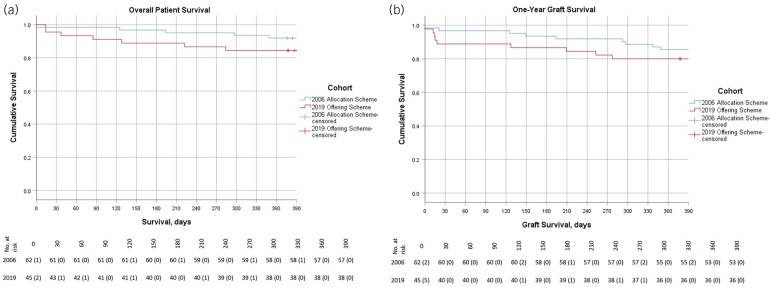
Kaplan–Meier graphs and life tables for (a) one-year patient survival and (b) one-year graft survival.

Kaplan–Meier graphs and survival tables for one-year patient and graft survival are demonstrated in Figures 2a and 2b, respectively. The one-year overall patient survival was 92% in the 2006 allocation scheme group and 84% in the 2019 offering scheme group (*p* = 0.214). The one-year graft survival was 85% in the 2006 allocation scheme group and 80% in the 2019 offering scheme group (*p* = 0.402). There were seven (6.5%) early graft losses, two from the 2006 scheme and five from the 2019 scheme. Both grafts from the 2006 allocation scheme and three from the 2019 offering scheme were lost because of surgical complications relating to the KT; the other two were deaths indirectly related to the KT. Three (2.8%) patients died within 30 days of KT. One patient died of complications relating to COVID-19 within the first year following KT.

### Univariable binary logistic regression analysis

A univariable binary logistic regression analysis was performed to compare the two groups (Table 5). KT recipients in the 2019 offering scheme were more likely to have undergone a previous KT (OR 4.691, 95% CI 1.385–15.893, *p* = 0.013). Induction immunosuppression with alemtuzumab was significantly less likely within the 2019 offering scheme group (OR 0.357, 95% CI 0.158–0.807, *p* = 0.013). The likelihood of a level 4 HLA mismatch was four times greater in the 2019 offering scheme group (OR 4.667, 95% CI 1.640–13.275, *p* = 0.004). In the 2019 offering scheme older adult KT recipients were statistically more likely to require critical care admission (OR 5.619, 95% CI 1.448–21.812, *p* = 0.013) and re-intervention (OR 6.496, 95% CI 1.306–32.216, *p* = 0.022). Finally, CMV viraemia was significantly more likely within the 2019 offering scheme group (OR 5.149, 95% CI 1.820–14.567, *p* = 0.002).

**Table 5:** Univariable binary logistic regression analysis comparing the 2006 allocation scheme cohort and the 2019 offering scheme cohort

Variable	Odds ratio	95% confidence interval	*p*-value
Caucasian ethnicity	5.083	0.509–50.720	0.166
Previous transplantation	4.691	1.385–15.893	**0.013**
Dialysis vintage, months	1.002	0.990–1.014	0.737
Age of donor, years	1.024	0.995–1.054	0.101
Cold ischaemia time	1.001	1.000–1.003	0.065
Level 4 HLA mismatch	4.667	1.640–13.275	**0.004**
LOS, days	1.024	0.971–1.080	0.378
Induction immunosuppression, alemtuzumab	0.357	0.158–0.807	**0.013**
Critical care admission	5.619	1.448–21.812	**0.013**
Re-intervention	Alemtuzumab	Alemtuzumab	**0.022**
DGF	1.393	0.579–3.350	0.459
Graft biopsy	1.548	0.650–3.687	0.323
Acute rejection	2.333	0.765–7.113	0.136
CMV viraemia	5.149	1.820–14.567	**0.002**
Readmission	1.944	0.892–4.240	0.095
Patient survival at one year	2.100	0.621–7.105	0.233
Graft survival at one year	1.472	0.533–4.068	0.456

CMV = Cytomegalovirus; DGF = delayed graft function; HLA = human leucocyte antigen; LOS = length of stay

Statistically significant *p*-values are shown in bold.

## Discussion

### Understanding the impact of HLA mismatches

Favourable HLA-matching of donors and recipients is associated with improved graft survival and fewer episodes of acute rejection.^22,23,24,25^ In the absence of a living donor, waiting times for a compatible deceased donor can be lengthy, meaning recipients remain on dialysis for prolonged periods.^26^ Furthermore, the positive effects associated with HLA-matching may be confounded by many other characteristics such as CIT, induction immunosuppression, recipient age and donor age.^26^ A balance must be struck between the availability of well-matched kidneys and the risks associated with long-term dialysis.

The 2019 offering scheme exhibited increased rates of KT in difficult-to-match groups such as BAME patients and those undergoing re-transplantation. It is more difficult to find favourable HLA matches for patients from BAME backgrounds because of differences in the HLA types expressed in those populations who are under-represented in the donor pool.^27^ Patients undergoing re-transplantation are more likely to be highly sensitised with consequent higher rates of graft loss.^28^ Older adults undergoing re-transplantation are likely to have a long history of immunosuppression, multiple comorbidities and multiple previous surgical interventions, making them challenging recipients.^29,30^

Although the 2019 offering scheme successfully increased rates of KT in these groups, it was also associated with a fourfold greater likelihood of having a level 4 HLA mismatch. Under the 2006 allocation scheme, patients with high matchability scores would not have received named offers in the context of a level 4 mismatch and would have been less likely to undergo KT.^13^ Permitting level 4 mismatches within the 2019 offering scheme appears to have positively influenced rates of KT in BAME and highly sensitised groups, in part addressing such disadvantages in the 2006 offering scheme. It would be beneficial to examine future KT to determine whether our findings persist beyond the bedding-in period of the new scheme as the inequality is addressed.

During the COVID-19 pandemic, modifications were made to induction immunosuppression regimes used in the 2019 offering scheme group. Alemtuzumab, a monoclonal antibody targeting CD52, normally used for immunologically challenging transplants was used less commonly because of its cytotoxic effect on lymphocytes.^31^ Owing to the increased risk of infection, basiliximab, a monoclonal antibody that competitively inhibits interleukin-2-mediated activation of lymphocytes, was preferred,^32^ thus introducing a lack of standardisation between regimes. It is therefore difficult to fully understand the impact of the less-favourable HLA-matching on episodes of acute rejection and graft survival.

Increased rates of acute rejection were observed in the 2019 offering scheme, although these were not statistically significant. The changes in immunosuppression protocols, described above, could have impacted this. Increased rates of CMV viraemia were seen in the 2019 offering scheme group, perhaps because of the enhanced immunosuppression required to treat acute rejection.

### Postoperative complications and survival

The 2019 offering scheme was associated with higher rates of re-intervention and critical care admission, reflecting the difficulties associated with transplantation of ECD kidneys, and the medical complexity of comorbid, potentially frail recipients.^33,34^ Recipients experienced complications requiring re-intervention both directly and indirectly related to their KT. We chose to include complications indirectly related to KT as they are indicators of frailty (eg sequelae of falls and confusion) and age-related surgical complexity (e.g. previous operations and difficult anatomy). This study showed an increase in the number of ECD transplantations in the 2019 offering scheme despite a more cautious approach to KT during the COVID-19 pandemic.^35^ We believe our data support the hypothesis that the change in the allocation system has meant that older recipients are more likely to be offered kidneys from higher risk donors leading to increased perioperative morbidity. This situation is unlikely to change given the discrepancy between the number of patients on the waiting list and the availability of kidneys for transplantation. Therefore, older recipients require further counselling on the risks of KT and a proactive approach to shared decision making.

One-year patient survival decreased from 92% to 84%, although this was not statistically significant and was confounded by a small sample size. The NHS Blood and Transplant Annual Report on Kidney Transplantation 2022/2023 quotes overall one-year deceased donor KT patient survival as 96% (95% CI 96–97).^36^ Data are not reported by age-group, making further comparison difficult. We postulate that with an increased sample size and further follow-up data points, there may be a statistically significant difference between the groups. Future application of robust frailty assessment, as described by the ongoing Kidney Transplant In Older People study, may prove a useful adjunct to age stratification alone.^17^

CIT was higher in the 2019 offering scheme group, although did not reach significance in the univariable analysis. Increased CIT has been associated with DGF and decreased graft survival, potentially contributing to less-favourable outcomes in the 2019 offering scheme cohort.^37^ Higher CIT within this group can be expected given the regionalisation of DCD offers in the 2019 allocation scheme leading to longer travel times, especially in the northern region, which is the largest geographical area, encompassing northern England, Scotland and North Wales.^14^

### The role of frailty in KT

Frailty is a multifactorial condition leading to increased vulnerability to physiological stressors with associated impaired ability to maintain homeostasis following insult.^38,39^ Increasing age is closely associated with increasing frailty burden.^40^ Frailty assessment tools are often not validated in patients on KRT. Nevertheless, frailty is an important consideration in the work-up for KT because an estimated 16% of recipients are frail before KT.^4,17^ At new KT assessment clinic, we have introduced frailty screening using the Clinical Frailty Scale.^41^ This supports clinicians in identifying patients who would benefit from assessment by a specialist frailty physician with surgical expertise, assisting in shared decision making and counselling on the risks associated with KT in older adults.

In KT, frailty has been associated with DGF, a LOS >2 weeks and early hospital readmission.^5,42^ In this study, the readmission rate was 60% during the 2019 allocation scheme. Readmission places a significant burden on patients, impacting quality of life and risking further complications associated with hospital stay such as nosocomial infections.

### Study limitations

Patients may have presented to local hospitals with complications and therefore are not included in this study. Clinically significant issues would likely be discussed with, or transferred back to, the transplanting centre, mitigating potential under-reporting.

The COVID-19 pandemic put a hold on KT in the UK. This resulted in a reduction in the number of KT recipients eligible for analysis in the 2019 offering scheme group and may have influenced patient selection and immunosuppression, confounding the results.

The single-centre retrospective nature of this study means it is at risk of selection bias. The univariable logistic regression model was not taken forward into a multivariable analysis due to sample size, limiting the power of the statistical analysis. This study would benefit from an independent external dataset to validate the reported findings.

## Conclusions

The introduction of the 2019 offering scheme has led to less-favourable HLA-matching but higher rates of KT in difficult-to-match groups such as BAME and re-transplantation in older recipients. The scheme was associated with a greater need for re-intervention and critical care admissions in KT recipients ≥60 years old. The use of a frailty assessment tool could facilitate the identification of older patients at risk of adverse outcomes following KT and support preoperative patient-centred risk–benefit counselling.

## Data Availability

Individual requests for data will be considered on a case-by-case basis.
